# Serum heavy metals and hemoglobin related compounds in Saudi Arabia firefighters

**DOI:** 10.1186/1745-6673-4-18

**Published:** 2009-07-07

**Authors:** Abdulrahman L Al-Malki

**Affiliations:** 1 Biochemistry Department, Faculty of Science, King Abdualziz University, Jeddah, Kingdom of Saudi Arabia

## Abstract

**Background:**

Firefighters are frequently exposed to significant concentrations of hazardous materials including heavy metals, aldehydes, hydrogen chloride, dichlorofluoromethane and some particulates. Many of these materials have been implicated in the triggering of several diseases. The aim of the present study is to investigate the effect of fire smoke exposure on serum heavy metals and possible affection on iron functions compounds (total iron binding capacity, transferrin saturation percent, ferritin, unsaturated iron-binding capacity blood hemoglobin and carboxyhemoglobin,).

**Subjects and methods:**

Two groups of male firefighter volunteers were included; the first included 28 firefighters from Jeddah city, while the second included 21 firefighters from Yanbu city with an overall age rang of 20–48 years. An additional group of 23 male non-firefighters volunteered from both cities as normal control subjects. Blood samples were collected from all volunteer subjects and investigated for relevant parameters.

**Results:**

The results obtained showed that there were no statistically significant changes in the levels of serum heavy metals in firefighters as compared to normal control subjects. Blood carboxyhemoglobin and serum ferritin were statistically increased in Jeddah firefighters, (p < 0.05 and p < 0.05 respectively) and Yanbu firefighters, (p < 0.005 and p < 0.001 respectively) as compared to normal control group while serum TIBC and UIBC were statistically decreased in Yanbu firefighters as compared to Jeddah firefighters, (p < 0.005 and p < 0.005 respectively) and normal control group, (p < 0.005 and p < 0.01 respectively). On the other hand, serum transferrin saturation percent was elevated in only Yanbu firefighters, (p < 0.05) as compared to Jeddah firefighters. Besides, there was no statistically significant change in blood hemoglobin and serum iron on comparison between all studied groups.

**Conclusion:**

Such results might point to the need for more health protective and prophylactic measures to avoid such hazardous health effects (elevated Blood carboxyhemoglobin and serum ferritin and decreased serum TIBC and UIBC) that might endanger firefighters working under dangerous conditions. Firefighters must be under regular medical follow-up through standard timetabled medical laboratory investigations to allow for early detection of any serum biochemical or blood hematological changes.

## Background

Fire Smoke is produced by either Combustion oxidation or pyrolysis [1]. Smoke may also contain characteristic trace and heavy elements such as lead, boron, cadmium, selenium, arsenic, antimony and molybdenum [2].

Lead is a multitargeted toxicant, affecting the gastrointestinal tract, hematopoietic, cardiovascular, central and peripheral nervous systems, kidneys, immune, and reproductive systems. As for arsenic, acute inhalation exposures can damage mucous membranes, causing rhinitis, pharyngitis and laryngitis. Chronic inhalation exposures can lead to rhino-pharyno-laryngitis, tracheobronchitis [3]; dermatitis, hyperpigmentation, and hyperkeratosis leucopenia, peripheral nerve dysfunction, [4] and peripheral vascular disorders [5]. Toxicity resulting from chronic exposure to mercury usually affects the kidneys and/or nervous system. Inhalation exposure to cadmium may result in headache, chest pains, muscular weakness, pulmonary edema and death. Renal toxicity may also result from inhalation exposure to cadmium, [6]. There is limited evidence from epidemiologic studies for cadmium-related respiratory tract cancer. Long-term occupational exposure to antimony has resulted in electrocardiac disorders, respiratory disorders, and possibly increased mortality [7].

Carbon monoxide is a narcotic compound that is responsible for up to 80 percent of fire related fatalities. Lethal concentrations of carbon monoxide are generally attained within l-3 hours of initiation of smouldering combustion. Inhaled carbon monoxide combines with the hemoglobin of red blood cells. The reaction of carbon monoxide with hemoglobin yields carboxyhemoglobin which is inactive in oxygen transport since both gases react with the same group in the hemoglobin molecule. The decrease in oxygen transport capacity is proportional to the percentage of carboxyhemoglobin [8].

Measurements of serum iron and total iron binding capacity are widely used in the diagnosis and treatment of iron deficiency anemia and chronic inflammatory disorders [9]. The clinical assessment of iron stores relied on the determination of serum iron, total iron-binding capacity and percent transferrin or direct examination of bone marrow [10].

The first aim of the present study is to investigate if heavy metals found in fire are transferred to the firefighters' body, and secondly, the impact of fire smoke exposure on serum iron and related compounds (Serum Iron, Total Iron Binding Capacity, Transferrin saturation percent, Ferritin, Unsaturated Iron-Binding Capacity Blood Hemoglobin and Carboxyhemoglobin,)

## Subjects and methods

The study protocol was approved by the local ethics committee. A written informed consent was obtained from all subjects. Two groups of male non-smokers firefighters volunteered to participate in the study: The first included 28 firefighters from Jeddah, mean age and standard deviation (39 ± 6.5). The second included 21 firefighters from Yanbu, mean age (43 ± 7.5). An additional group of 23 male non-firefighters volunteered from both cities as normal control subjects, mean age (41 ± 7.3). All subjects were clinically investigated to exclude those who were suffering from acute and/or chronic illnesses(as hypertension, diabetic, cardiac or occupational diseses). In particular, normal chest x-ray was an essential exclusion clinical parameter for the normal control group. All firefighter volunteers were randomly chosen for participation regardless of the type of burning materials and scale of fire accidents they faced (household or industrial fire). An official coordination was arranged with the Civil Defense Administrations to obtain their consent to conduct the research, and all participants were informed well about the objective and the course of the study.

Ten milliliters of fasting venous blood were drawn from each participant of the normal control group and the two firefighters groups within the first hour of firefighting a fire accident, regardless of time, scale, or type of the fire accidents they faced; 5 ml of blood on lithium heparin for iron and related compounds and 5 ml of blood without anticoagulant for serum separation were also withdrawn.

### Determination of Serum Heavy Metal

Serum samples were lysed for analysis of heavy metals by adding 10 ml HNO_3 _to one ml of serum and heated for 3–4 hours, and then 1 ml perchloric was added to the same sample. Digestion process continued until the solution was clear. Atomic absorption spectrophotometry-flamless method was used to determine serum Lead, Cadmium and Antimony by using Shimadzu AA-6650G instrument with electronic double-beam Graphite Furnace Atomic Absorption, (GFAA) Spectrophotometer.

Atomic absorption spectrophotometry-Hydride Vapor Generator method was used to determine serum arsenic and serum mercury [10,11] by using Shimadzu AA-6650F.

### Determination of Serum Iron and Some of its Biologically Active Derivatives

Serum Iron, (Fe) was determined according to [12]. Serum Total Iron Binding Capacity, (TIBC) [13]. Serum Transferrin saturation percent, (%TS) = (Serum Iron/TIBCx100), [14]. Serum Ferritin [15]. Serum Unsaturated Iron-Binding Capacity, (UIBC) = Total Iron Binding Capacity (TIBC) – Serum Iron [16]. Blood Hemoglobin, (HGB) [17]. Blood Carboxyhemoglobin, (COHb) [18].

### Statistical Analysis

Statistical analysis was performed on a PC using SPSS, V.13. Data are presented as arithmetic mean ± S.D., with subsequent use of Student t-test for the determination of the significance of difference between sample means.

## Results

There was no statistically significant difference in serum heavy metals in Jeddah firefighters as compared to normal control group, Yanbu firefighters as compared to normal control group and Jeddah firefighters as compared to Yanbu firefighters respectively (Table 1, Table 2 and Table 3). Results presented in tables (Table 4, Table 5 and Table 6) show that blood carboxyhemoglobin, (COHb) and serum ferritin levels were statistically significantly elevated in Jeddah firefighters, (p < 0.005 and p < 0.05 respectively) and Yanbu firefighters, (p < 0.005 and p < 0.001 respectively) as compared to normal control group. On the other hand, serum total iron binding capacity, (TIBC) and unbound iron binding capacity, (UIBC) were statistically significantly elevated in Jeddah firefighters, (p < 0.005 and p < 0.005) and normal control group, (p < 0.005 and p < 0.01 respectively) as compared to Yanbu firefighters. However, serum transferrin saturation percent was statistically significantly decreased in Jeddah firefighters as compared to Yanbu firefighters, (p < 0.05) as shown in table 6. Statistical comparison between Jeddah and Yanbu firefighters showed that there were significant differences in TIBC, transferring and UIB.

**Table 1:** Statistical Analysis of Serum Heavy Metals in Jeddah Firefighters as Compared to the Normal Control Group, (mean ± S.D.).

**Parameter**	**Normal Control Group **	**n**	Jeddah FFs*	**n**	**t-test**	**p- value**
**Lead (μg/dL) **	3.73 ± 1.21	8	3.49 ± 1.06	8	0.4208	N.S.

**Arsenic (μg/dL)**	0.36 ± 0.12	8	0.34 ± 0.23	8	0.1898	N.S.

**Mercury (μg/dL)**	0.27 ± 0.04	8	0.27 ± 0.05	8	0.1520	N.S.

**Cadmium (μg/dL)**	0.08 ± 0.06	8	0.07 ± 0.03	8	0.2444	N.S.

**Antimony (μg/dL)**	0.00 ± 0.00	8	0.00 ± 0.00	8	----	N.S.

*FFs: firefighters

N.S: non-significant

**Table 2:** Statistical Analysis of Serum Heavy Metals in Yanbu Firefighters as Compared to the Normal Control Group, (mean ± S.D.).

**Parameter**	**Normal Control Group**	**n**	Yanbu FFs*	**n**	**t-test**	**p- value**
**Lead (μg/dL) **	3.73 ± 1.21	8	3.83 ± 1.64	16	0.14453	N.S.

**Arsenic (μg/dL)**	0.36 ± 0.12	8	0.33 ± 0.15	18	0.4464	N.S.

**Mercury (μg/dL)**	0.27 ± 0.04	8	0.28 ± 0.05	19	0.5200	N.S.

**Cadmium (μg/dL)**	0.08 ± 0.06	8	0.10 ± 0.08	18	0.7711	N.S.

**Antimony (μg/dL)**	0.00 ± 0.00	8	0.00 ± 0.00	18	----	N.S.

*FFs: firefighters

N.S: non-significant

**Table 3:** Statistical Analysis of Serum Heavy Metals in Yanbu Firefighters as Compared to Jeddah Firefighters, (mean ± S.D.).

**Parameter**	Jeddah FFs*	**n**	Yanbu FFs*	**n**	**t-test**	**p- value**
**Lead (μg/dL) **	3.49 ± 1.06	8	3.83 ± 1.64	16	0.52231	N.S.

**Arsenic (μg/dL)**	0.34 ± 0.23	8	0.33 ± 0.15	18	0.1267	N.S.

**Mercury (μg/dL)**	0.27 ± 0.05	8	0.28 ± 0.05	19	0.3283	N.S.

**Cadmium (μg/dL)**	0.07 ± 0.03	8	0.10 ± 0.08	18	1.0303	N.S.

**Antimony (μg/dL)**	0.00 ± 0.00	8	0.00 ± 0.00	18	----	N.S.

*FFs: firefighters

N.S: non-significant

**Table 4:** Statistical Analysis of Serum Iron and Some of its Biologically Active Derivatives in Jeddah Firefighters as Compared to the Normal Control Group, (mean ± S.D.).

**Parameter**	**Normal Control Group **	**n**	Jeddah FFs*	**n**	**t-test**	**p- value**
**HGB (g/dl)**	15.52 ± 1.51	23	16.26 ± 1.12	27	1.9801	N.S.

**COHb (%)**	3.000 ± 1.27	17	5.43 ± 2.91	22	3.20993	p < 0.005

**Iron (ug/dl) **	86.43 ± 25.76	21	80.0 ± 28.84	28	0.821094	N.S.

**TIBC (ug/dl)**	324.36 ± 33.99	22	330.48 ± 50.77	27	0.4833	N.S.

**Transferrin sat. (%)**	26.31 ± 9.83	22	24.06 ± 9.40	27	0.8175	N.S.

**Ferritin (ng/ml)**	78.19 ± 34.09	20	123.26 ± 63.10	27	2.89175	p < 0.05

**UIBC (ug/dl)**	240.64 ± 49.45	22	253.30 ± 61.27	27	0.7830	N.S.

*FFs: firefighters

N.S: non-significant

**Table 5:** Statistical Analysis of Serum Iron and Some of its Biologically Active Derivatives in Yanbu Firefighters as Compared to the Normal Control Group, (mean ± S.D.).

**Parameter**	**Normal Control Group **	**n**	Yanbu FFs*	**n**	**t-test**	**p- value**
**HGB (g/dl)**	15.52 ± 1.51	23	15.66 ± 1.55	21	0.3040	N.S.

**COHb (%)**	3.000 ± 1.27	17	4.93 ± 2.37	21	3.01979	p < 0.005

**Iron (ug/dl) **	86.43 ± 25.76	21	87.52 ± 27.15	21	0.1341	N.S.

**TIBC (ug/dl)**	324.36 ± 33.99	22	283.38 ± 50.34	21	3.1421	p < 0.005

**Transferrin sat. (%)**	26.31 ± 9.83	22	31.31 ± 9.57	21	1.6908	N.S.

**Ferritin (ng/ml)**	78.19 ± 34.09	20	169.55 ± 103.22	14	3.70087	p < 0.001

**UIBC (ug/dl)**	240.64 ± 49.45	22	195.91 ± 50.76	21	2.9270	p < 0.01

*FFs: firefighters

N.S: non-significant

**Table 6:** Statistical Analysis of Serum Iron and Some of its Biologically Active Derivatives in Yanbu Firefighters as Compared to Jeddah Firefighters, (mean ± S.D.).

**Parameter**	Jeddah FFs*	**n**	Yanbu FFs*	**n**	**t-test**	**p- value**
**HGB (g/dl)**	16.26 ± 1.12	27	15.66 ± 1.55	21	1.5493	N.S.

**COHb (%)**	5.43 ± 2.91	22	4.93 ± 2.37	21	0.6138	N.S.

**Iron (ug/dl) **	80.00 ± 28.84	28	87.52 ± 27.15	21	0.9396	N.S.

**TIBC (ug/dl)**	330.48 ± 50.77	27	283.38 ± 50.34	21	3.2003	p < 0.005

**Transferrin sat. (%)**	24.06 ± 9.40	27	31.31 ± 9.57	21	2.6333	p < 0.05

**Ferritin (ng/ml)**	123.26 ± 63.10	27	169.55 ± 103.22	14	1.78416	N.S.

**UIBC (ug/dl)**	253.30 ± 61.27	27	195.91 ± 50.76	21	3.4641	p < 0.005

*FFs: firefighters

N.S: non-significant

## Discussion

Most important of all are the poisonous effects of heavy metals. Firefighters are the subjects most exposed to toxicants that may have adverse effect on their life. Two groups of firefighters from Jeddah and Yanbu cities and one control group were included in this study.

Results presented in tables (1 &2 &3) showed that there were no statistically significant changes in the levels of serum heavy metals between firefighters and control group. This is in accordance with the study of [19], which stated that mercury levels were not higher in exposed firefighters but are mentioned because of heightened concern about exposure at the World Trade Center. One control and three exposed firefighters had total blood mercury levels > 20 μg/l, a conservative upper reference limit. Because blood inorganic mercury was < 1.7 μg/l for all exposed firefighters, these elevated total blood mercury concentrations represent organic mercury contributions from dietary sources, (e.g., fish consumption) rather than from exposure.

The urinary antimony-adjusted geometric mean of the Special Operations Command group was two times higher than that of the other exposed firefighters or controls [20]. Two populations (firefighters and the general population) were surveyed in four cities for urine heavy metal concentrations. Arsenic and cadmium levels were significantly related to smoke exposure, and for firefighters, arsenic levels were significantly related to exposure [21].

Measurements of serum iron and total iron binding capacity are widely used in the diagnosis and treatment of iron deficiency anemia and chronic inflammatory disorders [22]. Historically the clinical assessment of iron stores has relied on the determination of serum iron, total iron-binding capacity and percent transferrin or direct examination of bone marrow. The literature suggests that ferritin provides a more sensitive, specific and reliable measurement for determining iron deficiency at an early stage [23].

Results of statistical analysis of serum iron and its related reactive derivatives in table (4) showed that blood carboxyhemoglobin and serum ferritin were statistically increased in Jeddah firefighters, (p < 0.05 for each) and Yanbu firefighters, (p < 0.005 and p < 0.001 respectively) as compared to normal control group while serum TIBC and serum UIBC were statistically decreased in Yanbu firefighters as compared to Jeddah firefighters, (p < 0.005 for each) and normal control group, (p < 0.005 and p < 0.01 respectively). On the other hand, serum transferrin saturation percent was elevated in only Yanbu firefighters, (p < 0.05) as compared the Jeddah firefighters, there was no statistically significant change in blood hemoglobin and serum iron comparing all studies group.

Exposure to carbon monoxide is determined by the measurement of the percent carboxyhemoglobin, (%COHb). The brain and the heart may be severely affected after CO exposure with carboxyhemoglobin, (COHb) levels exceeding 20%, [24], although earlier studies in coal mine fires indicated that very few victims had 60% COHb at death, almost all having ≥ 80 COHb. Fire victims could have less and sometimes much less than 50% COHb, yet their death can be clearly attributed to smoke inhalation from other evidence obtained at autopsy and other investigations [25].

Levy [26] stated that, a statistically significant difference was found between the mean baseline carboxyhemoglobin of non-smoking firemen and smoking firemen. A consistent increase in mean COHb levels after exposure to smoke was seen in both non-smoking and smoking men, but the mean increase in these two groups was statistically significant only at the 90 percent level, (t = 1.85, p < 0.1).

This is in accordance with our results obtained in table (4). Kales [27] conducted an investigation of unexpectedly high level carboxyhemoglobin in a group of firefighters. Twelve of 34, (35%) nonsmokers tested had levels greater than 4% COHb and 9 of 34, (26%) had levels of 10% or higher. All 24 nonsmoking firefighters retested had COHb levels less than 3%. Baseline carboxyhemoglobin readings of 64 firefighters ranged from 0% to 3% (mean 1% and median 1%). One hundred eighty-four carboxyhemoglobin readings were collected during training exercises. The mean and median carboxyhemoglobin levels were 1%. The maximum value in a firefighter wearing self-contained breathing apparatus was 3%; values of 14%, 5%, and 4% were measured in instructors who were not properly wearing self-contained breathing apparatus [28-30].

## Conclusion

Such results might point to the need for more health protective and prophylactic measures in order to avoid such hazardous health effects that might endanger firefighters working under highly dangerous conditions. Firefighters must be under regular medical follow-up through standard timetabled medical laboratory investigations to allow for early detection of any hematological changes.

## Competing interests

The author declares that they have no competing interests.
